# The Tendency to Lean on the Past

**Published:** 1898-09

**Authors:** 


### Editorial.

### THE TENDENCY TO LEAN ON THE PAST.

CONSERVATISM is an excellent brake in the chariot of progress, and serves as a regulator in all the relations of life, but a dependence upon it is as injurious as the hasty advance from old things to new without due consideration.

The progress of dentistry has not been checked, in any large degree, by precedent. The profession is entirely too new to have the old lines so well marked that any overstepping leads to criticism. While this is true there is a tendency to regard innovations with something of the prejudice characteristic of established custom which has so marked all the various phases of human endeavor.

The time has been when dentistry was a law unto itself. It absorbed, both in its theoretical and practical, all possible from older thoughts and practice, and that without much regard to methods adopted in the schools or in the laboratories. It has also marked out for itself an honorable share of original research; in fact, it is to that it owes its present position as one of the very important factors of modern civilization.

The fifty years of experience has led by slow degrees to a broader curriculum in all the dental schools, and now, in the last days of the century, these stand, as a rule, fully abreast with that of medicine.

With this advance of the standard of training there has been developed a tendency to lean upon the past and allow the mind to rest satisfied with the conclusion that there is nothing more to be gained not already known. This is the insidious poison that has weakened not only professions but peoples.

It requires but a slight familiarity with the nationalities of Europe to perceive that there is in everything there a continued and close adherence to precedent. The marked contrast between the rapid progress in this country, as compared with the slower evolutions there, has been noticed and commented upon, but the lessons taught in the war at present upon us should not be lost in this direction. The United States government marked out for itself its own course of procedure in both arms of the national service, which, as it failed to follow European ideas, was met, as we know, by the contemptuous criticism of all military and naval experts. The result has fully justified the departure from established usage, yet, while it has silenced criticism, it is not probable it will change the methods of foreign countries. These are fixed, and it will require something more than the present war to change them. It remains, however, one of the most valuable object-lessons, and serves to illustrate the necessity of freedom in thought and practice, if progress in any direction is to be maintained.

It has frequently been expressed upon these pages that law within certain limits is not only necessary but valuable as a mental stimulus to correct thinking, but, carried beyond healthy bounds, becomes a weakening force, tending to a dependence upon power, and landing nations into the conservative quicksands from which there is no escape. All people necessarily retrograde slowly but surely as they come to depend upon law and the mediocrity in thought which it engenders. When freedom of thought and action has ceased in America, as at present in Europe, through conservative influences, that will mark the period of its decadence.

This truth, as applied to nations, is more forcibly pronounced when considered in connection with professions. Here the conservative element is ever in the ascendant and becomes a continual bar to progress. The natural tendency of professional education is to confirm this feeling in the younger element, and it is feared will have a retarding influence in the near future. It has learned too much to depend on authority, and the dictum of the college hall will remain with them as the standard for many years to come. This is one of the injurious effects of high training, and should be met by radical treatment during the undergraduate period. The imperative need of the hour is to combat this inertia by instilling into the students of our dental colleges an enthusiasm for original investigations in all lines of our work. This is not difficult of accomplishment if the teacher is earnestly interested. His enthusiasm will soon strike a responsive chord in many minds. To meet this properly there must be less of didactic teaching in our colleges and more direct contact with the pupil by the teacher. It is not probable that the former method will be dispensed with, as there is no better way to instil principles, but the magnetism of direct contact must be more generally introduced if the dental profession is to continue the progressive body it has been in the past.

The world needs to get away from the idea that what is good enough for us is sufficient for our children. Every generation needs something better than the preceding. Perfection has never been reached in any direction, and it is safe to assume will not at any time in the future history of civilization.

There was a painful illustration of the tendency to lean upon the past when the new national organization was completed at Old Point Comfort. Before this reaches our readers this body will have met and will, probably, continue upon the old lines. Conservatism conquered, and the effect, it is feared, will be disastrous to all true progress. We need new life, new measures, new men, if we are to reach out and grasp the embryo thoughts of the coming time. The crutches of the past must be thrown aside and dentistry must walk erect in its own paths. If any change be made in the next few years in the machinery of our work, it is hoped a careful consideration will be given to the needs of the present, with a due regard to the possible wants of the future.

The organization of societies should be based upon this view. Constitutions of fifty years ago should be permitted to lie in the literary mausoleums of the past. Let the new wine be placed in new bottles labelled for use in the generation producing it. Constitutions based on immutable principles can never change, but many, like old creeds, need constant revivifying to keep pace with the ever-growing thought of the time. There is such a thing as the antique in the formulation of the moral code, but there can never be antiquity in the essentials of ethics. We change the form of our dress, but the basic principle of clothing the human body has remained the same since man covered himself with skins.

Whatever moral force the conservative element in human nature possesses, it must not be permitted to shackle progress, and by progress is not meant change for the sake of change, but that thought should be rehabilitated to new conditions.

The old standards are giving way before greater enlightenment, and they are wise who rise up to meet these changes, not in an antagonizing spirit, but with the glad thought that every step made means one degree nearer civilization and one remove from the barbarism of prehistoric man.

